# Evolutionary Influences on Attribution and Affect

**DOI:** 10.3389/fpsyg.2017.02255

**Published:** 2017-12-22

**Authors:** Jennie Brown, David Trafimow

**Affiliations:** ^1^Department of Psychology, Franklin Pierce University, Rindge, NH, United States; ^2^Department of Psychology, New Mexico State University, Las Cruces, NM, United States

**Keywords:** social cognition, attribution, gender differences, reaction times, morality attribution, ability attribution, evolution

## Abstract

Evolutionary theory was applied to Reeder and Brewer's schematic theory and Trafimow's affect theory to extend this area of research with five new predictions involving affect and ability attributions, comparing morality and ability attributions, gender differences, and reaction times for affect and attribution ratings. The design included a 2 (Trait Dimension Type: HR, PR) × 2 (Behavior Type: morality, ability) × 2 (Valence: positive, negative) × 2 (Replication: original, replication) × 2 (Sex: female or male actor) × 2 (Gender: female or male participant) × 2 (Order: attribution portion first, affect portion first) mixed design. All factors were within participants except the order and participant gender. Participants were presented with 32 different scenarios in which an actor engaged in a concrete behavior after which they made attributions and rated their affect in response to the behavior. Reaction times were measured during attribution and affect ratings. In general, the findings from the experiment supported the new predictions. Affect was related to attributions for both morality and ability related behaviors. Morality related behaviors received more extreme attribution and affect ratings than ability related behaviors. Female actors received stronger attribution and affect ratings for diagnostic morality behaviors compared to male actors. Male and female actors received similar attribution and affect ratings for diagnostic ability behaviors. Diagnostic behaviors were associated with lower reaction times than non-diagnostic behaviors. These findings demonstrate the utility of evolutionary theory in creating new hypotheses and empirical findings in the domain of attribution.

## Evidence of evolutionary influences on social cognition

Evolutionary theory is a powerful paradigm that is becoming increasing useful in its applications to a wide variety of fields such as medicine/health (Nesse and Stearns, 2008), conservation (Bonduriansky et al., 2012), agriculture (Baucom and Holt, 2009), crime (Quinsey, 2002), politics (Kerr, 2002), art (Miller, 2000), literature (Gotschall and Wilson, 2005), music (Brown, 2000; Wallin et al., 2001), and psychology (Buss, 2009). Social psychology has especially benefitted from an evolutionary perspective (Buss, 2005; Gangestad and Simpson, 2007; Crawford and Krebs, 2008). However, evolutionary theory has not fully made its way into social cognition and attribution. The purpose of this experiment was to test new predictions, using evolutionary theory, in attribution.

Evolutionary theory provides a useful explanation of why humans distinguish between different types of behavior and resulting trait attributions. Evolutionary theorist MacLean (1983) argued that humans evolved from being somewhat solitary creatures to social creatures. Being a solitary creature, one would not need to worry a great deal about the character of others. However, as humans became more social, knowing the character of others became more important to navigate the social scene successfully. Avoiding individuals who possessed potentially harmful traits would be very advantageous.

### Attribution theory

Compared to other attribution theories such as adaptation level theory (Helson, 1964), social judgment theory (Sherif and Sherif, 1967), correspondent inference theory (Jones and Davis, 1965), novelty theory (Fiske, 1980), and range theory (Birnbaum, 1972; Wyer, 1973, 1974); Reeder and Brewer's (1979) schematic theory makes the most precise predictions and can account for the greatest amount of phenomena/data where trait attribution is concerned. This theory predicts what types of behavior should lead to strong correspondent trait attributions and what types of behavior should not. It also accounts for the differences in attributions and behavior expectations for ability and morality behaviors/traits and accounts for negativity and positivity biases. It predicts when information other than the observed behavior will be considered in attribution. Finally, this theory has been supported by a great deal of empirical evidence (Reeder et al., 1977; Reeder and Spores, 1983; Reeder and Coovert, 1986; Reeder, 1993, 1997; Trafimow and Schneider, 1994; Trafimow, 1997; Trafimow and Trafimow, 1999; Brown et al., 2004; Trafimow et al., 2005). In sum, Reeder and Brewer's (1979) schematic theory not only addresses the problems the other theories do not, it is also able to account for the same data for which other theories account. See Table 1 for synopsis of schematic theory qualities.

**Table 1:** Schematic theory qualities.

	**Positive morality**	**Negative morality**	**Positive ability**	**Negative ability**
**PARTIALLY RESTRICTIVE DIMENSIONS**				
Behavior is perceived to be diagnostic of actor's trait	No	No	No	No
Predicted bias	No	No	No	No
Predicts only correspondent expectancies	No	No	No	No
Predicts both correspondent and non-correspondent expectancies	Yes	Yes	Yes	Yes
Only the observed behavior is important in considering attribution	No	No	No	No
Information about the situation, actor and behavior frequency are important in considering attribution	Yes	Yes	Yes	Yes
**HIERARCHICALLY RESTRICTIVE DIMENSIONS**				
Behavior is perceived to be diagnostic of actor's trait	No	**Yes**	**Yes**	No
Predicted bias	No	**Yes**	**Yes**	No
Predicts only correspondent expectancies	Yes	**No**	**No**	Yes
Predicts both correspondent and non-correspondent expectancies	No	**Yes**	**Yes**	No
Only the observed behavior is important in considering attribution	No	**Yes**	**Yes**	No
Information about the situation, actor and behavior frequency are important in considering attribution	Yes	**No**	**No**	Yes

*Bold indicates, highlight the distinctiveness of diagnostic behaviors as compare to non-diagnostic behaviors*.

Reeder and Brewer's schematic theory (1979) argues that the attribution process involves the distinction between two types of trait dimensions, partially restrictive (PR) and hierarchically restrictive (HR). With this distinction, it is possible to predict what information will carry the most weight in the attribution process as well as which behaviors will result in trait attributions.

PR trait dimensions have two characteristics. First, neither positive nor negative behaviors are diagnostic of an actor's character, and thus PR related behaviors do not elicit strong trait attributions. For example, if an actor performed an unkind behavior (negative PR behavior), an observer would not be confident that this one behavior was indicative of the actor's character—the observer would not be sure that the actor was an unkind person (see Table 2 for example behaviors). The second characteristic is that situational information^1^ is important in attributing behavior. The observation of an unkind behavior may be attributed to factors other than the actor's trait possession. The behavior may have occurred because of the actor's mood (a bad mood could lead to the exhibition of an unkind behavior) or external pressures (the target of the unkind behavior is generally unkind, which lead the actor to reciprocate). Also, an observer may need to see many PR behaviors to believe that the behavior is evidence of the actor's trait possession.

**Table 2:** Behavioral stimuli based on category—ability/morality, diagnostic/non-diagnostic.

**DIAGNOSTIC**			
HR Ability	Intelligent	Being goal-oriented in his/her career	Voicing his/her opinion and backing it with evidence
	Creative	Creating a new comedy skit for an upcoming talent show	Creating a new style of clothing
HR Morality	Dishonest	Stating that you truly believe that being a Nazi is wrong when you yourself are one^*^	Cheating on an exam^*^
	Disloyal	Ditching a friend because other people think he/she is weird, etc.^*^	Disclaiming your family because you think they are an embarrassment^*^
**NON-DIAGNOSTIC**			
HR Ability	Unintelligent	Not trying to learn anything new	Using the restroom in public rather than finding a restroom
	Uncreative	Buying an outfit because someone on TV was wearing it	Not making an effort to make projects/ presentations stand out
HR Morality	Honest	Tell a guy/girl you are “not interested” from the start, instead of leading them on^*^	Not plagiarizing schoolwork^*^
	Loyal	Putting family before friends or fun^*^	Being true to your kids—if you tell them you'll do something, do it^*^
PR Ability	Sociable	Being interested in what other people have to say	Not behaving shyly around new people
	Unsociable	Interrupting someone when he/she is trying to talk	Belching out loud in a restaurant
	Studious	Studying for exams sooner than the night before	Asking teacher for clarification in lecture
	Unstudious	Not taking notes in class	Not looking at or studying for Spanish until the night before the exam
PR Morality	Friendly	Speaking to the person who serves you coffee (or anything else) like a human being^*^	Talking to those who seem uncomfortable in an unfamiliar situation^*^
	Unfriendly	Ostracizing someone from the group^*^	Giving a rude response when asked a simple question^*^
	Charitable	Reading to the children at a library or hospital^*^	Buying a homeless person lunch^*^
	Uncharitable	Making a smart remark when asked to donate something to a group^*^	Getting a free car wash and not giving a donation^*^

* *From Chadwick et al. (2006)*.

HR trait dimensions differ from PR trait dimensions in two ways. First, HR trait dimensions are asymmetric, whereas PR dimensions are symmetric (both poles of the dimension are perceived to be equally indicative of an actor's character). For HR trait dimensions, positive and negative behaviors differ in their diagnosticity. Also, situational information is considered to affect attributions for some types of behavior, but not for all. Because these two characteristics differ depending on whether the behavior type is related to morality or ability, morality and ability dimensions will be discussed separately.

Where morality is concerned, the negative pole of the trait dimensions carries the diagnosticity (there is a negativity bias—negative information is given more weight in attributions). Some HR trait dimensions related to morality are honest-dishonest and loyal-disloyal. For example, if an actor were to perform a dishonest behavior, an observer would be confident that the actor is a dishonest person (and a trait attribution is made). But, if an actor performs an honest behavior, an observer would not be certain that the actor is an honest person (and a trait attribution is not made). HR dimensions also differ from PR dimensions in that situational information is not very important in the attribution process when an actor engages in a negative behavior, but this information is important when an actor engages in a positive behavior. If an actor engages in an honest behavior, observers perceive that the behavior may be due to reasons other than trait possession (it may have been beneficial to do so in that situation or she was in a good mood) and the frequency of the behavior is also considered (is the actor always honest or just this time?). However, if an actor performs one dishonest behavior, this is seen to be indicative of her trait possession regardless of situational information. It seems that there is no good reason for immoral (in this example, dishonest) behavior (for an exception see Brown et al., 2004).

Where ability is concerned, positive HR behaviors are diagnostic. If an actor demonstrates a positive HR behavior in the ability domain, observers are confident that the actor possesses the corresponding trait (this is referred to as a positivity bias—positive information is given more weight in attribution). Only someone possessing high ability can successfully perform a behavior requiring high ability. If an actor fails to demonstrate a positive HR behavior, observers are not confident that this is indicative of the actor's lack of ability. An example of an HR dimension related to ability are ability-inability to slam-dunk a basketball (Trafimow, 1997, 2001). If an observer sees an actor slam-dunk a basketball the observer will be confident that this is indicative of the actor's basketball ability, but when an observer sees an actor fail to make a slam-dunk, she is not sure whether the actor lacks the ability or not—even Michael Jordan missed occasionally.

In sum, for both ability and morality, both positive and negative PR behaviors are not considered to be diagnostic of an actor's character and situational information is an important consideration in the attribution process. However, diagnosticity, and the consideration of situational information differs when they correspond to HR trait dimensions. For morality, negative HR behaviors lead to strong trait attributions and the behavior is the most important factor in determining attributions (not situational information). Strong trait attributions are not made for positive behaviors and situational information is perceived to be important in making attributions. In the ability domain, the reverse is true. Observers make strong trait attributions for positive behaviors and they perceive situational information to be important in making attributions. Observers do not make strong trait attributions for negative behaviors and they do not consider situational information to be important.

HR and PR trait dimensions/behaviors may have differential importance where evolutionary theory is concerned. If a behavior is indicative of a trait and not due to situational factors that could potentially change, it is logical that humans would pay more attention to them and give more weight to them. These types of behaviors would give observers more information regarding the actor's character and would let observers know how to interact with the actor. Additionally, a dishonest or disloyal (diagnostic behaviors) friend or acquaintance could lead to the loss of resources or could damage other relationships. Loss of resources or damaged relationships could potentially lead to fewer offspring and/or death for an individual, especially in a harsh environment where resources are scarce. An individual who is merely unkind (non-diagnostic behavior) can be avoided and his behavior may not be as damaging as a disloyal or dishonest behavior. Also, an unkind behavior is immediately obvious, but dishonest or disloyal behavior may not be immediately obvious. The target of a dishonest or disloyal behavior may not discover that he is the target of such damaging behavior, or he may not discover it until he has already experienced the negative effects.

### Importance of morality

Thus far we have argued from an evolutionary point of view why observers should distinguish between negative HR and PR morality behaviors, but we have only touched on why traits related to morality may be more important than those related to ability. When one considers the ways in which a negative HR morality behavior could have important consequences for others, it is not difficult to imagine numerous circumstances in which the effects of such behavior could be harmful. But it is quite difficult to imagine situations in which an actor's positive HR ability behavior could have important consequences for others. This is especially the case when one considers that positive HR ability behaviors rarely, if ever, target an individual; but negative HR morality behaviors always target others. For example, when an actor is dishonest and lies to another person, this other person is the target, but when a person slam-dunks a basketball, the effects are not nearly as important for others.

Philosophers have historically made similar arguments for behaviors/traits in the domain of morality. Kant (1797/1991) developed the Categorical Imperative which is comprised of rules for moral guidance about what people *should* do. According to Kant, “perfect duties” are perfect in that they allow for no exceptions regardless of a person's mood, situation, or any other consideration. Honestly is an example of a perfect duty and according to Kant individuals should never lie. However, imperfect duties are conditional. Individuals can violate imperfect duties from time to time, perhaps to fulfill other imperfect duties, if they are obeyed some of the time. So, an individual may refuse to give money to a charity so that she can use the money to buy a gift for a friend. According to Kant, imperfect duties are duties of virtue. Fulfilling them results in merit for the actor, but not fulfilling them does not result in blame.

Trafimow and Trafimow (1999) found that Kant's distinction between perfect and imperfect duties corresponded to the distinction participants make between HR and PR morality trait dimensions and provided an a priori principle by which traits/behaviors could be categorized as HR or PR. In their experiments, they found that participants changed their positive expectancies about an actor's trait possession more quickly because of a perfect duty violation rather than an imperfect duty violation. They also found that situations affect attributions for the violation of imperfect duties but not perfect duties.

Not only did Kant (1797/1991) argue that some negative behaviors are more important than others, Aristotle (c. 330 BC [1982]) argued that some negative behaviors were especially damaging to relationships (such as dishonesty and disloyalty, negative HR behaviors/perfect duty violations). Although this phenomenon has been argued by philosophers and empirically supported by researchers, it is not clear *why* this phenomenon exists. Reeder and Brewer (1979) have done an excellent job of explaining and demonstrating the phenomena that people distinguish between different types of behaviors/traits. However, they have not described the underlying reason why observers possess these schemas that make some traits/behaviors more important than others.

### The role of affect

Kant (1797/1991) argued that the process by which individuals distinguish perfect from imperfect duties is a rational process that requires much cognitive work and philosophical sophistication. However, participants appear to have no trouble making Kant's distinction and it is doubtful that many of them have had any or much logical/philosophical training. Thus, there must be a less effortful process or at least a process that uses very few cognitive resources. One plausible process by which participants could distinguish between HR and PR trait dimensions is affect. Johnston (1999) argued that affect offers humans meaning in life. Affect has also been found to be a strong factor in determining attitudes, which in turn is the best predictor of behavioral intentions and behavior (Mann, 1959; Ostrom, 1969; Abelson et al., 1982; Breckler, 1984; Millar and Tesser, 1986; Breckler and Wiggins, 1989; Pfister and Bohm, 1992; Crites et al., 1994; Eagly et al., 1994; Trafimow and Sheeran, 1998; Trafimow et al., 2004). If affect is such a powerful force in providing us with meaning and is such an important determinant in human behavior, it could very well be that affect helps determine the way in which we interpret the behavior of others.

In arguing that affect is what drives trait attribution, there are two assumptions that must be addressed. First, when considering morality there should be more negative affect because of a negative HR behavior than that experienced resulting from a negative PR behavior. Second, the degree of the negative affect experienced in response to a negative HR behavior should be greater than the positive affect experienced when observing positive HR and all PR behaviors. These assumptions change when considering ability. With ability, there should be more positive affect resulting from a positive HR behavior than that experienced resulting from positive PR behavior. Also with ability, the degree of positive affect experienced in response to a positive HR behavior should be greater than the negative affect experienced when observing negative HR and PR behavior. In sum, greater affect should be experienced when observing positive HR ability behavior and negative HR morality behavior than other types of behavior. Trafimow et al. (2005) tested the hypothesis that affect is the driving force in morality related attributions. In five studies, they demonstrated not only an association between affect and attribution, but also a *causal* relationship. This finding makes a compelling case for the causal role of affect in trait attributions—but only where morality is concerned.

Although Trafimow et al. (2005) makes a compelling case for the role of affect in morality attributions, it does not address the role, if any, of affect in ability attributions. There are two reasons why affect is likely to be involved in ability attributions. First, if affect provides meaning in life (Johnston, 1999), is a major determinant in our behavior, and has been shown to be causally related in attributions of morality (Trafimow et al., 2005), then it seems likely that it would be a causal factor in ability attributions as well. Second, there is a great deal of evidence, although anecdotal/non-empirical, that humans experience positive affect when observing others exhibit positive HR ability behaviors. For example, many people all over the world spend their resources to observe actors exhibiting positive, HR ability behaviors. Individuals pay a great deal of money to observe people exhibiting their high ability in such behaviors as singing in rock concerts and opera (this is comparable to singing a Mozart aria—Trafimow, 1997, 2001), slam-dunking a basketball (Trafimow, 1997, 2001) or many other feats of ability (many people watch/attend professional/college athletic events and the Olympics). It appears to be the case that observers are willing to *lose* resources that could have been used for many other different purposes to gain some positive affect. The effects of this positive affect are visible in the behavior of the observers: they smile, cheer and clap; sometimes they even shed tears of happiness or jump up and down. Although this evidence is anecdotal and not empirical, it is quite compelling and can be tested empirically.

If affect is to be included in a theory for understanding the attribution process, it is desirable to show that it is causally related to ability attributions as well as to morality attributions. Because this distinction is the clearest with HR dimensions, only behaviors pertaining to HR dimensions will be discussed in the remainder of this section. The affect experienced by observers in response to positive ability behaviors may be less than that experienced because of negative morality behaviors since it may not have important consequences (this is discussed below). These less important or unimportant consequences should lead to slightly weaker trait attributions as well. There is some evidence suggesting that attributions are more extreme for negative morality related behavior than for positive ability related behavior (Skowronski and Carlston, 1987). Although unrelated to the purposes of their study, data collected by Skowronski and Carlston (1987) indicate that negative morality related behaviors were rated more negatively than positive ability behaviors were rated positively. Also, the range of attributions for morality behaviors is wider than the range for ability behaviors. There are several reasons to suppose that ability and morality attributions should have such relationships. First, morality attributions are potentially more personally relevant than ability attributions. Morality related behavior frequently targets other individuals, whereas ability related behavior does not. Second, the consequences of a negative morality behavior are far more important than those of a positive ability behavior.

### Gender differences

If the affect that drives attribution is the result of evolution creating a more adaptive way of interacting with others, it may be that evolution shaped our attribution processes differentially according to gender. Evolutionary psychologists have argued that evolution has led to differences in male and female thought and behavior. One such difference is evident in human sexual strategies (Bateman, 1948; Williams, 1966; Trivers, 1972; Symons, 1979; Daly et al., 1982; Draper and Harpending, 1982, 1988; Daly and Wilson, 1983; Buss, 1989, 1994; Tooby and Cosmides, 1989; Kenrick et al., 1990; Clutton-Brock and Vincent, 1991; Cronin, 1991; Buehlman et al., 1992; Buss et al., 1992; Baker and Bellis, 1993; Buss and Schmidt, 1993; Simpson et al., 1993; Walsh, 1993; Ridley, 1994). Typically, men seek women who are young, attractive (which is highly indicative of health/fertility), and faithful (at least when long-term relationships are being considered) (Malinowski, 1929; Morris, 1967; Trivers, 1972; Symons, 1979; Shostak, 1981; Daly et al., 1982; Daly and Wilson, 1983; Buss et al., 1992; Buss, 1994), whereas women seek men who are not only attractive (which is highly indicative of health/fertility) but also resourceful (Buss, 1989, 1994; Tooby and Cosmides, 1989). While youth and attractiveness are not considered morality or ability traits, faithfulness, and resourcefulness are. Therefore, it may be more important for *females* to be moral than it is for males. (We are not arguing that it is unimportant for males to be moral, just that it may be more important for females). This prediction can be supported by jealousy research, which has demonstrated that males fear physical infidelity more so than females (Trivers, 1972; Symons, 1979; Daly et al., 1982; Buss et al., 1992). Thus, honesty and loyalty should be traits that are especially important for females to possess. In fact, when providing resources for offspring, males depend on the loyalty and honesty of females to ensure that these resources are not squandered on someone else's genetic material (someone else's offspring) unknowingly. If this is the case, when participants observe a female perform a negative HR morality behavior, their attributions should be more negative than attributions for males who perform the same type of behavior. Also, participants should experience more negative affect in response to the female's behavior than they experience in response to the same type of behavior by a male.

Conversely, it may be the case that when participants are rating *male* actors, ability may be more important than it is when considering female actors. Even now, high ability can translate into greater resources (especially where HR traits are concerned). In our evolutionary past, ability would have been quite important for the survival of its possessor, his mate(s) and offspring. Greater resourcefulness would have been translated into greater access to mates; those who were less resourceful would have had fewer opportunities to reproduce. Furthermore, females who did not seek males who were resourceful would not have been as successful in passing on their genes. Thus, resourcefulness, an ability trait, should be a very important characteristic in evaluating males. If this is true, males who demonstrate a positive HR ability behavior should be rated more positively than females who engage in the same type of behavior.

There is another piece of evidence that suggests that ability may be more important for males than females. In the recent past, men's athletics have received much more funding and other types of support (more fans/spectators and more television air time) at all levels (middle school, high school, college, and professional) than women's. In fact, Title IX was enacted to alleviate this problem. Furthermore, women's athletics (in general) has been only a recent invention, whereas men's athletics has a very long history (even cheerleading and gymnastics—which are now considered predominately female activities were originally participated in by males only).

### Hypotheses:

H_Morality_–Participants' attributions and affect for morality related behaviors should be stronger than attributions and affect for ability related behaviors.

H_Ability_–Participants' attributions for HR ability behaviors should be highly related to the affect they experience in response to these behaviors.

H_Female_–For negative HR morality behaviors, participants' attributions will be stronger for female actors than for male actors.

H_Male_–For positive HR ability behaviors, participants' attributions will be stronger for male actors than for female actors.

H_Time_–Participants' attributions for negative HR morality and positive HR ability behaviors will take less time to make than attributions for the other types of behavior (positive HR morality, negative HR ability, and all PR behaviors).

## Method

### Participants

One hundred and twenty-five undergraduate students from New Mexico State University and El Paso Community College voluntarily participated in this experiment to partially fulfill course credit or for extra credit. The mean age was 21.99 (Mdn = 20.00, SD = 6.00) and there were 80 females and 47 males. Fifty-five percent of participants were Latino, 35% Caucasian, 5.8% Black, and 8.9% categorized as other/missing.

### Design

All of the hypotheses were tested in a 2 (Trait Dimension Type: HR, PR) × 2 (Behavior Type: morality, ability) × 2 (Valence: positive, negative) × 2 (Replication: original, replication) × 2 (Sex: female or male actor) × 2 (Gender: female or male participant) × 2 (Order: attribution portion first, affect portion first) mixed design. All factors were within-participants except the order of attribution and affect measures (participants were randomly assigned to do one or the other first) and the gender of the participant. The within participants design of this experiment was implemented for two reasons; first, this allowed the researchers to maximize the number of participants, and second, this minimized error variance associated with a between participants design.

### Stimuli

Before testing these hypotheses, there were two issues to address. One issue to resolve was whether to use concrete or abstract behaviors as stimuli. Typically, behaviors that serve as stimuli in studies of ability attribution are concrete, whereas morality attribution studies use abstract behaviors. By using abstract behaviors, researchers do not need to worry about whether different behaviors are equivalent because all the information is the same except the behavior. However, a major criticism of attribution research where morality is concerned is that in the “real world” individuals do not encounter abstract behaviors. Therefore, attributions in response to abstract behaviors may not be truly indicative of the attributional process. For these experiments, we used concrete behaviors.

The second issue to resolve was how to compare ability and morality attributions directly. This required obtaining a set of both types of behaviors that have been equated on *Z*-scores (otherwise it would be like comparing apples and oranges). For negative morality and positive ability behaviors to be compared, we needed to match them in perceived strength (not valence). For example, a dishonest behavior needs to be perceived to be as dishonest as a slam-dunk is perceived to be indicative of basketball ability. Chadwick et al. (2006) tested a wide range of morality related behaviors to obtain a list of behaviors which vary in perceived morality. By obtaining a similar list of behaviors that pertain to ability rather than morality, we will be able to choose ability and morality behaviors that have similar Z-scores and can be directly compared.

Sixteen morality related behaviors were selected from Chadwick et al. (2006) and 16 ability behaviors were obtained using a similar process described in the Chadwick article. This resulted in two behaviors from the following categories (32 behaviors overall): intelligent, unintelligent, creative, uncreative, sociable, unsociable, studious, unstudious, honest, dishonest, loyal, disloyal, friendly, unfriendly, charitable, and uncharitable behaviors (see Table 2 for list of behaviors by category). For each of these behavior categories, one was assigned to a female actor and one for a male actor. These behaviors were chosen based on three criteria: (1) they must have been likely to be performed by both male and female actors, (2) each behavior was required to have received a *Z*-score close to 0.5, and (3) each behavior was required to have a standard deviation <1^2^. Behaviors were assigned to male and female actors in such a way that *Z*-score values (although very close to 0.5) were approximately equally distributed across the ability/morality, male/female, HR/PR, and diagnostic/non-diagnostic categories.

After behaviors were assigned to each of the categories they were developed into short scenarios. Different male and female names were assigned to the actors in each scenario so participants would not assume that the same actor was performing each behavior. The 16 most frequently occurring male names and the 16 most frequently occurring female names in the U.S. (according to the 1990 U.S. Census) were chosen (Most Common Names in the United States, 1998). An example of one of these scenarios was: “James voiced his opinion and backed it with evidence” (an intelligent item). E-prime (Schneider et al., 2001) was used to present stimuli and measure attribution and affect ratings and the corresponding reaction times.

### Procedure

When participants arrived to participate in the experiment they were asked to sit in front of a computer and read and complete a consent form. After signing the consent form, they were orally given a brief set of instructions by the experimenter. Participants were assigned to one of two conditions: (1) affect first/attribution second or (2) attribution first/affect second, based on the number they were assigned (even numbered participants were in Condition 1 and odd numbered participants were in Condition 2).

For the affect portion of the experiment, participants were presented with two slides (screens) consecutively. The first slide contained the scenario. Once the participant read the scenario, he was instructed to “press the spacebar to continue.” This action initiated the appearance of the affect response slide. This slide asked the participant, “How does (actor's) behavior make you feel.” Below this on the same slide was a seven-point bipolar scale. The scale appeared as seven text boxes. Above text box one, on the left was the text *extremely pleasant*. Above box seven on the right was the text *extremely unpleasant*. To make a rating choice, the participant used the mouse to click on one of the boxes. After a response was selected, the next scenario slide was presented, followed by an affect response slide. Each participant received all 32 scenario slides and affect response slides in a random order. In addition to the affect measure, reaction times were also taken for each affect measure. Timing began when the participant pressed the space bar (after reading the scenario slide) and ended when the participant made a choice on the affect response slide. Participants could not move on to a new scenario until they made an appropriate response. If a participant clicked on a portion of the screen other than one of the 7 text boxes, they were presented with the same scenario slide followed by the response slide.

The attribution phase proceeded in the same manner. The participant was presented with one of the scenario slides (the same that were used in the affect portion). After reading the slide and pressing the space bar, they were presented with the corresponding attribution response slide. This slide read, “Rate (actor name)” and was also followed by a 7-point bipolar scale. Each attribution response slide corresponded to the scenario in that the extremes referred to the trait that the behavior was supposed to be representing. For example, when a participant received the scenario, “James voiced his opinion and backed it with evidence” it would be followed by an attribution response slide that ranged from *extremely intelligent* to *extremely unintelligent*. As with affect response slides, the participant would not be presented with a new scenario until he had made an appropriate response. If the participant clicked on an inappropriate portion of the screen, the same scenario slide and attribution response slide would be presented again. In addition to recording attribution responses, attribution reaction times were taken in the same way that they were taken for affect responses.

After completing the affect and attribution portions of the study, the participants were presented with a screen that read “Contact Experimenter.” The participant was then thanked for his participation, debriefed, and excused from the experiment.

## Results

Results will be discussed in terms of their relevance to the five hypotheses. Although the within participants nature of this experiment allows for the examination of many interaction effects, only main effects will be discussed as hypotheses were only relevant to main effects.

H_Morality_ predicted that participants' attributions and affect for morality related behaviors would be stronger than attributions and affect for ability related behaviors. Before testing this hypothesis, we needed to standardize the attributions and affect ratings in two steps. First, to ensure a true zero point for participants, we standardized the scores by subtracting each attribution rating from the grand mean (0.33 SD = 2.15) of all the attribution ratings. This process was repeated for the affect ratings (grand mean = 0.22, SD = 1.94). Second, we took the absolute value of all the items so that items could be combined and compared directly to other items. Prior to this, it was noted that the affect and attribution ratings were in the “right” direction—negative behaviors were given negative attributions and affect ratings and positive behaviors were given positive attributions and affect ratings. After standardizing the data in this way, a within-participants planned comparison was used to test for differences between attributions for ability and attributions for morality. The mean attribution rating for ability behaviors was 1.74 (SD = 0.52) and the mean rating for morality was 2.14 (SD = 0.45) and this difference was significant, *t*_(124)_ = 46.18, *p* < 0.001, *d* = 0.82. This analysis was repeated for affect ratings for morality and ability. The mean affect ratings for ability was 1.35 (SD = 0.58) and for morality was 1.88 (SD = 0.54) and this difference was also significant, *t*_(124)_ = 9.58, *p* < 0.001, *d* = 0.94.

H_Ability_ predicted that participants' attributions for HR ability behaviors should be highly related to the affect they experience in response to these behaviors. Support for this hypothesis was obtained by examining both within- and between-participants correlations for attribution and affect responses for HR ability behaviors. The mean within-participants correlation for all ability attributions was *r*_(123)_ = 0.65, which was significantly different from 0, *t* = 36.33, *p* < 0.001. The between-participants correlation was also significant, *r*_(123)_ = 0.38, *p* < 0.001. For HR ability behaviors only, the mean within-participants correlation for ability attributions was *r*_(123)_ = 0.65, *t* = 38.97, *p* < 0.001. The between-participants correlation was also significant for this comparison, *r*_(123)_ = 0.29, *p* < 0.001. For PR ability behaviors only, the mean within-participants correlation was *r*_(123)_ = 0.57, *t* = 24.42, *p* < 0.001. With this comparison (only PR ability behaviors) the between-participants attribution-affect correlation was also significant, *r*_(123)_ = 0.30, *p* < 0.001.

H_Female_ predicted that for negative HR morality behaviors, participants' attributions would be stronger for female actors than for male actors. A planned comparison revealed that there was a significant difference, *t*_(124)_ = −4.75, *p* = 0.001, *d* = 0.37. The mean attribution for female actors who engaged in negative HR behaviors was 2.65 (SD = 0.96) and the corresponding mean attribution for male actors was 2.29 (SD = 0.98). This finding suggests that participants make stronger attributions for females who engage in negative HR morality related behavior than for males who engaged in the same type of behavior.

The H_Male_ hypothesis proposed that attributions for positive HR ability behaviors should be stronger for male actors than for female actors. A planned comparison revealed that the difference between attributions for male and female actors was not significant. The mean attribution for males for ability was 1.88 (SD = 0.94) and the mean for females for ability was 1.83 (SD = 0.90), *t*_(123)_ = 0.858, *p* = 0.393, *d* = 0.37. In sum, the H_Male_ hypothesis was not supported; participants did not make significantly different attributions for males and females where ability is concerned.

The final hypothesis H_Time_ posited that participants' attributions for negative HR morality and positive HR ability (diagnostic) behaviors will take less time to make than attributions for other types of behavior (non-diagnostic—positive HR morality, negative HR ability, and all PR behaviors). Since reaction times may vary greatly from participant to participant, these scores were standardized for each participant. For each participant, the mean of his or her reaction times for attribution items was subtracted from each of his or her attribution reaction times to produce standardized attribution reaction times. This was repeated for affect reaction times using the mean affect reaction time. After standardizing the times in this way, a within-participants planned comparison revealed that there was a significant difference in reaction times for the two categories of behaviors in the predicted direction. The mean reaction time for diagnostic behavior attributions and the other attribution reaction times were 2918.67 (SD = 745.82) ms and 3327.98 (SD = 248.61) ms, respectively, *t*_(123)_ = −1.99, *p* < 0.001, *d* = 0.74. However, this comparison for affect reaction times was not significant. Reaction times for affect responses were lower for diagnostic behaviors (positive HR ability/negative HR morality) than non-diagnostic behaviors but this difference was not significant [*M* = −776.93, SD = 611.02 and *M* = −854.67, SD = 203.67 ms, respectively], *t*_(123)_ = −0.835, *p* < 0.41, *d* = 0.17. Thus, this hypothesis was partially supported in that attribution reaction times were significantly lower in response to diagnostic behaviors than reaction times non-diagnostic behaviors. However, this difference was not significant for affect reaction times.

## Discussion

The purpose of this research was to test five novel hypotheses by applying evolutionary theory/logic to the attribution process. The hypotheses received mixed support from the data. The first hypothesis argued that morality related behaviors are more important than ability behaviors. If this is so, actors who engage in morality related behaviors should receive stronger attributions and affect ratings than actors who engaged in ability related behaviors. This hypothesis was supported. The second hypothesis predicted that affect would play a role in attributions for HR ability behaviors as well as HR morality behaviors^3^. This hypothesis was supported. Now affect can be included as part of a general attribution theory since we know it is involved in both morality attributions and ability attributions. The female hypothesis was supported. Females were given stronger negative attributions and affect ratings than males after performing negative HR morality behaviors. The male hypothesis was not supported. Males and females were given similar attribution and affect ratings for performing positive HR ability related behaviors. The final hypothesis was that reaction times would be much faster for diagnostic behaviors (positive HR ability and negative HR morality) than for non-diagnostic behaviors. This hypothesis was also supported. Overall, four hypotheses were supported (H_Morality_, H_Ability_, H_Female_, and H_Time_) and one was not (H_Male_).

The findings for the time hypothesis were surprising—the reaction times for attribution ratings were in the predicted direction, but the reaction times for the affect ratings were not. There are two possibilities when considering the role of affect in attributions (where reaction times are concerned). One possibility is that when a participant encounters a diagnostic behavior it causes a strong affective reaction, which causes him to react quickly—making a quick attribution. However, when a participant encounters a non-diagnostic behavior he experiences a weak affective reaction and must think about what type of attribution to make. This possibility is supported by the data. It seems that affect mediates the participant's need to think (making him think then make an attribution or not think and just make an attribution). Thus, attribution reaction times are faster for behaviors that participants do not need to think about, but the reaction times are slower when participants do need to think about the behavior.

This experiment was different from previous attribution studies in several ways. First, this was the first *extensive* within-participants study of attribution. In Trafimow and Trafimow (1999), participants were asked to make multiple attribution judgments in a within-participants design, but it was not as extensive as the current experiment. Participants in Trafimow and Trafimow (1999) made four attributions and in the present experiment participants made 32 attributions. The application of an evolutionary perspective generated several new predictions. This was the first experiment to directly compare ability and morality attributions and to make and test predictions about the relationships between them. This was also the first experiment to examine the role of affect in ability attributions. Another first was the examination of the differences in attributions and affect according to the sex of the actor. Previous attribution and person memory studies examined attributions (and occasionally affect) for male actors only. Finally, reaction times have very rarely been used to study the attribution process and when they were used, they were used in a different way (see Uleman et al., 1992).

Because this study was different from other attribution studies in so many ways, there were many new findings. Although these findings were new, they did not contradict schematic theory but rather extend it. First, schematic theory does not make predictions about the roles of affect, the gender of the actor, reaction times, or whether participants differ in their attributions/affect for morality and ability behaviors. However, by applying evolutionary theory to schematic theory we could make and test new predictions using schematic theory.

One important finding is that affect matters for ability attributions as well as morality attributions. Trafimow et al. (2005) made a compelling case for the role of affect in morality attributions, but they did not address the role of affect in ability attributions. Affect can now be included in a theory for understanding the attribution process, including how affect differs for ability and morality behaviors. The findings supported our argument that the affect experienced by observers in response to positive ability behaviors would be less than that experienced because of negative morality behaviors. We argued that this would be the case because morality related behavior frequently targets other individuals, whereas ability related behavior does not, and because morality related behaviors have more important consequences than ability related behaviors. This finding is consistent with Peeters' distinction between “good and bad” traits for others (other-profitability) vs. traits for the self (self-profitability)^4^. In this experiment, participants rated an actor's traits and not their own, thus they were thinking about how the actor's traits would affect those who interact with the actor (in our experiment the “targets”). In three of Peeter's experiments, the key traits for positive self-profitability were more consistent with ability traits (ambitious, brilliant, self-confident), while key traits for negative other-profitability were more in line with morality (selfish, unreliable, aggressive, intolerant; Peeters, 1992, 2001; Peeters et al., 1998). As Peeter's work predicts, our participants found the other-profitability traits more important (given strong affect and attribution ratings) for the actor to possess than self-profitability traits.

Trafimow (1997) argued that ability attributions are a result of a cognitive process by which observers judge the likelihood that an actor could perform a behavior given his ability level. For example, the likelihood of an actor successfully slam-dunking a basketball (positive HR ability behavior), if he did not possess high ability in slam dunking is very low (maybe impossible). Conversely, the possibility of an actor successfully making a free throw (positive PR ability behavior) is somewhat likely even if he did not possess high free throwing ability. So, behaviors that are highly unlikely (if the actor does not possess the ability) are being much more diagnostic of the actor's ability, and thus lead to stronger attributions. But behaviors that are somewhat likely even if the actor does not possess the ability are not believed to be as diagnostic and do not lead to strong attributions. When these findings are taken together with the findings of the present study, that attributions in response to positive HR ability behaviors take much less time than attributions in response to the other types of behaviors, the attribution picture becomes more interesting. It may be that this perception of the probability of behaviors may determine how much time it takes to make an attribution. So, behaviors that are highly unlikely for actors without the requisite ability may take less time to consider. Observers know that this highly unlikely behavior means that the actor possesses the ability. However, behaviors that are more likely to be exhibited (even if the actor does not possess the ability) take more time to consider. Making an attribution takes more time because the observer is not sure what this means for the actor's ability. Thus, the current study does not contradict Trafimow (1997); it is an extension of it.

The findings regarding reaction time to affect strength were surprising. There are two possibilities when considering the role of affect in attributions (where reaction times are concerned). One possibility is that when a participant encounters a diagnostic behavior (positive HR ability, negative HR morality), it causes a strong affective reaction, which causes them to react quickly. This results in an attribution being made quickly. When a participant encounters a non-diagnostic behavior (like studious or friendly, etc.) they experience a weak affective reaction and they must think about what type of attribution to make. The second possibility is that diagnostic behaviors result in a fast affective reaction which leads to a fast attribution. However, when a participant encounters a non-diagnostic behavior he has a slow affective reaction and an attribution is made slowly. If this second possibility is correct, then the affect reaction time for diagnostic behaviors should be significantly faster than the affect reaction time for non-diagnostic behaviors. If the first possibility is correct, then there would be no effect for affect reaction times. This second possibility is supported by the data. It seems that affect mediates the participant's need to think (making him think then make an attribution or not think and just make an attribution). Thus, attribution reaction times are faster for behaviors that participants do not need to think about, but the reaction times are slower when participants do need to think about the behavior.

Additionally, the H_Ability_ hypothesis extends Trafimow (1997) in another way. According to H_Ability_, attributions should be related to the affect experienced by participants in response to HR ability behaviors. If diagnostic behaviors are so unlikely that attributions are made quickly, it may be that they are also made with little or no effort. It could be that affect is the shortcut by which these probability judgments are made. It is difficult to determine what causes what in the ability attribution process (about reaction times, affect or probability judgment). It may be that greater affect makes the observers feel that the behavior is highly unlikely, or it may be that the probability is so ridiculously low that observers react more affectively. Future research could test these possibilities.

Evolutionary theory has been valuable in understanding and predicting social behavior and this experiment demonstrates this. Thus far, evolutionary theorizing has not been extended to the realm of social thought—particularly attribution. However, when “evolutionary logic” was applied to the attribution process it yielded several interesting and testable predictions. First, is it more important to be moral or to have high ability? Ability could be important in social situations. If you possess high ability, you can impress your friends and gain their respect and maybe even acquire more resources for yourself and others. However, being moral may be much more important, as this type of behavior more directly affects others on a personal level. Morality related behavior targets other individuals, whereas ability related behavior does not. At best, someone's high ability behavior will benefit others, if he can acquire resources because of his ability AND is willing to share (which brings us back to a morality issue). As humans evolved from being solitary creatures to social creatures (MacLean, 1983), knowing the character of others became more important. In our evolutionary past when resources were scarce, the behavior of others could be the difference between life and death. An immoral friend/acquaintance could lead to the loss of resources or damage to other relationships. This could potentially lead to fewer offspring, loss of social support, or death of an individual.

The second prediction derived from evolutionary theory was that it may be more important for females to be moral than for males. Research has demonstrated that males fear physical infidelity more so than females (Trivers, 1972; Symons, 1979; Daly et al., 1982; Buss et al., 1992), thus honesty and loyalty should be traits that are especially important for females to possess. In the past, men depended on women to be loyal and honest to ensure that their children really were their own. Thus, it would make sense that morality is perceived to be more important for females than for males to possess and this is just what the data suggest. It may be that the need for females to be honest and loyal is so strong that this expectancy spills over into areas unrelated to sexual fidelity. Perhaps observers feel that if a female is dishonest or disloyal in one area, she is likely to dishonest/disloyal in others.

Another issue that could be addressed in a future experiment would be an extension of the male and female hypotheses. We mentioned in the introduction that literature suggests that males look for females who are loyal and honest (in addition to other qualities) and females look for males who are resourceful (in addition to other qualities) (Bateman, 1948; Williams, 1966; Trivers, 1972; Symons, 1979; Daly et al., 1982; Draper and Harpending, 1982, 1988; Daly and Wilson, 1983; Buss, 1989, 1994; Tooby and Cosmides, 1989; Kenrick et al., 1990; Clutton-Brock and Vincent, 1991; Cronin, 1991; Buehlman et al., 1992; Buss et al., 1992; Baker and Bellis, 1993; Buss and Schmidt, 1993; Simpson et al., 1993; Walsh, 1993; Ridley, 1994). It would be interesting to test these ideas more specifically. For example, if an actor performs an act of sexual infidelity, it may not matter whether the actor is male or female. If the morality domain is specific to sex, maybe male and female actors will receive similar attributions. However, this would be contradictory to jealousy research (Trivers, 1972; Symons, 1979; Daly et al., 1982; Buss et al., 1992) and the present finding that morality is more important for females than males.

In regard to the current findings about males' ability behavior, it may be that ability behaviors need to be more specific to resourcefulness for a gender difference to be detected (if there is one). The current research suggested that ability is not more important for judging male behavior. However, overall ability behaviors received weaker attribution and affect ratings than morality behaviors. Therefore, it may be that this is a weaker effect and with a more targeted behavior there may be a gender difference.

## Conclusion

The present experiment was designed to test five hypotheses, four of which were supported with one disconfirmation. It would be tempting to argue that the one disconfirmation provides strong evidence against the theories from which they were derived. However, two considerations suggest that this would not be a convincing argument. This failure was in the predicted direction, though not statistically significant. Trafimow (2003) has demonstrated the problems with using *p*-values to draw conclusions about hypotheses. The other consideration is that given that the theories have performed well in the past, it seems more likely that the failure was caused by faulty auxiliary assumptions rather than by bad theories. As Lakatos (1970, 1978) pointed out, wrong hypotheses can be attributed to theories or to auxiliary assumptions, and for theories that have been strongly corroborated by surviving previous destructive tests (Popper, 1968), it often makes sense to put the blame on the auxiliary assumptions. Of course, if the auxiliary assumptions are supported by future research, or if the theories make wrong predictions when combined with other auxiliary assumptions, then it will be more reasonable to blame empirical failures on the theories. Although philosophers often talk about supporting or disconfirming theories, much work in science is devoted to “puzzle solving” (Kuhn, 1971). The present research is a case in point. For example, the present data indicate that behaviors pertaining to morality cause more affect than do behaviors pertaining to ability, and cause more extreme attributions to be made. None of the foregoing theories make this prediction, though the prediction is certainly consistent with them. Thus, the present data should be considered as an extension of previous work rather than as providing definitive tests of any theories. Perhaps, however, the present findings will lead to future revolutionary research.

## Ethics statement

This study was carried out in accordance with the recommendations of the U. S. Department of Health and Human Services (DHHS) regulations for the protection of human subjects [DHHS regulations for the Protection of Human Subjects (45 CFR 46)] with written informed consent from all subjects. All subjects gave written informed consent. The protocol was approved by the New Mexico State University Institutional Review Board (IRB).

## Author contributions

JB and DT made substantial contributions to the conception or design of the work; or the acquisition, analysis, or interpretation of data for the work; and drafted the work and revised it critically for important intellectual content; and offered final approval of the version to be published; and agreed to be accountable for all aspects of the work in ensuring that questions related to the accuracy or integrity of any part of the work are appropriately investigated and resolved.

### Conflict of interest statement

The authors declare that the research was conducted in the absence of any commercial or financial relationships that could be construed as a potential conflict of interest.

## Footnotes

^1^Situational information broadly refers to the situation in which the behavior occurs and includes the actor's mood, motivation, luck, effort, or the frequency of the behavior.

^2^*Z* = 0.5 was an arbitrary number.

^3^Recall that Trafimow (1997) provided support that affect was causally related to attributions of morality. The present experiment established that affect was related to ability attributions, but did not establish (or test for) a causal relationship.

^4^Self-profitability refers to the value of traits for the individual who possess these traits. Other-profitability refers to the value of an individual's traits to those who could potentially interact with that person. Other-profitability traits help individuals determine whether they should approach or avoid a particular actor (Peeters, 2001; Peeters et al., 2003).
